# Squeezed light from a nanophotonic molecule

**DOI:** 10.1038/s41467-021-22540-2

**Published:** 2021-04-14

**Authors:** Y. Zhang, M. Menotti, K. Tan, V. D. Vaidya, D. H. Mahler, L. G. Helt, L. Zatti, M. Liscidini, B. Morrison, Z. Vernon

**Affiliations:** 1 Xanadu, Toronto, ON Canada; 2 Department of Physics, University of Pavia, Pavia, Italy

**Keywords:** Quantum optics, Quantum information

## Abstract

Delicate engineering of integrated nonlinear structures is required for developing scalable sources of non-classical light to be deployed in quantum information processing systems. In this work, we demonstrate a photonic molecule composed of two coupled microring resonators on an integrated nanophotonic chip, designed to generate strongly squeezed light uncontaminated by noise from unwanted parasitic nonlinear processes. By tuning the photonic molecule to selectively couple and thus hybridize only the modes involved in the unwanted processes, suppression of parasitic parametric fluorescence is accomplished. This strategy enables the use of microring resonators for the efficient generation of degenerate squeezed light: without it, simple single-resonator structures cannot avoid contamination from nonlinear noise without significantly compromising pump power efficiency. We use this device to generate 8(1) dB of broadband degenerate squeezed light on-chip, with 1.65(1) dB directly measured.

## Abstract (web summary)

Integrated sources of nonclassical light are a key component for scalable quantum technologies. Here, the authors work with two coupled microring resonators and show how to detune the resonances involved in unwanted parametric fluorescence, without significantly affecting the pump power efficiency.

## Introduction

Squeezed light sources^1^ are a fundamental building block of photonic technologies for quantum information processing. Squeezing is an essential resource for quantum sensing^2^ and a wide range of quantum computing algorithms^3–6^. Recently, much effort has gone into engineering scalable implementations of such sources using integrated photonics. Compact and highly efficient sources of squeezing have been developed based on whispering gallery mode resonators^7^. Much progress has been made in developing squeezed light sources based on periodically poled waveguides in low-index-contrast platforms^8,9^. However, for applications requiring very large numbers of components, high-index-contrast nanophotonic platforms are preferred, as they enable large-scale integration with many hundreds or thousands of elements on a single monolithic chip^10^. Within the domain of nanophotonic structures, bright intensity-difference squeezing in a silicon nitride ring resonator driven above the parametric oscillation threshold has been demonstrated^11^. Subsequently, a two-ring structure was used to enable tuning of the level of squeezing by varying the effective resonator coupling condition^12^. Single-ring resonators driven below threshold have yielded two-mode (nondegenerate) quadrature squeezing and photon number difference squeezing^13^. Micromechanical resonators have generated small levels of squeezing over a few MHz of bandwidth^14^. Some squeezing in a single-mode degenerate configuration has also been reported with microrings using a single-pump^15^, but this suffers from strong excess noise contributions arising from non-parametric effects such as thermorefractive fluctuation^16^. Aside from limiting the amount of available squeezing, the presence of excess noise is especially undesirable for quantum computing applications, as such noise degrades the purity of the quantum states employed.

No nanophotonic device has yet been demonstrated which efficiently produces quadrature squeezed vacuum in a single, degenerate spectral mode, uncontaminated by excess noise from both non-parametric and unwanted parametric processes. A promising candidate has been proposed based on dual-pump spontaneous four-wave mixing in microring resonators^17,18^: two classical pumps tuned to independent resonances can produce squeezing in a single, degenerate spectral mode. Such resonators are also desirable for the broadband nature of the squeezing they can produce^19^. By using pumps very well separated in frequency from the squeezed mode, noise contributions from non-parametric effects like thermorefractive fluctuations can be avoided. However, in such a system a number of unwanted parametric effects^20^ add noise to the squeezing band, irreversibly corrupting the output. The primary culprit for such unwanted noise is single-pump parametric fluorescence^21^ driven by each individual pump; further degradation is caused by Bragg-scattering four-wave mixing^22^, which can transfer energy away from the squeezed mode.

The role of such parasitic parametric effects in degenerate squeezers based on single-resonator integrated structures was recently clarified by Zhao et al.^23^. A silicon nitride ring resonator was used to generate squeezing in a dual-pump configuration. Without suppression of parasitic effects, it was shown that only 0.8 dB of squeezing would have been achievable. To overcome this, some suppression of parasitic processes was achieved by detuning the pumps from resonance, with 1.34 dB of degenerate squeezing observed and 3.09 dB of squeezing inferred on-chip. However, this strategy suffers from a significant trade-off between squeezing and pump power efficiency, as detuning the pumps reduces their resonance enhancement in the ring, compromising the efficiency of the desired squeezing process.

In this work, we bypass the detuning approach by directly engineering a device that strongly suppresses unwanted parasitic nonlinear effects without significantly compromising generation efficiency. We leverage a design based on photonic molecules: These devices are composed of two or more optical resonators, arranged such that some of the modes of each resonator are coupled to those of the other. Such structures have been used for emulating the behavior of two-level systems^24^, lasing^25^, and on-demand optical storage and retrieval^26^. Coupled resonators have also been used for dispersion engineering of integrated devices^27,28^, enhancing their performance for nonlinear optical applications.

**Fig. 1:**
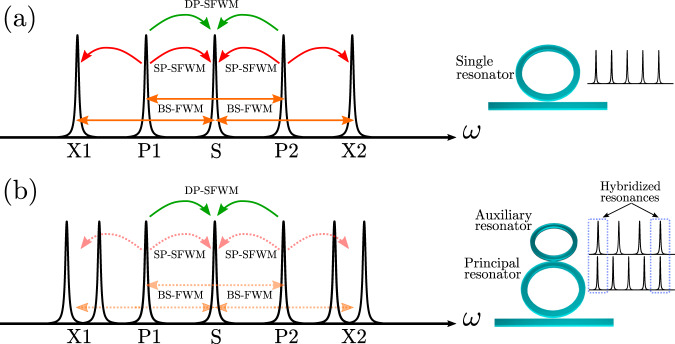
Resonance structure. **a** Idealized intensity enhancement inside a single-ring resonator, showing the four-wave mixing processes that occur when two resonances P1 and P2 are pumped. **b** Idealized intensity enhancement of the two-ring photonic molecule, showing the splitting and detuning of the hybridized X1 and X2 resonances that arises from the strong linear coupling between the principal and auxiliary resonator. In both cases the desired process, dual-pump spontaneous four-wave mixing (DP-SFWM, shown in green), leads to squeezing of the S mode. The unwanted processes of single-pump spontaneous four-wave mixing (SP-SFWM, red) generate excess noise in the S mode, contaminating the output, while Bragg-scattering four-wave mixing (BS-FWM, orange) transfers photons away from the S mode as photons are exchanged between the two pumps. Both these unwanted processes are suppressed by the presence of the auxiliary resonator, without significantly affecting the desired squeezing process. The free spectral ranges of the auxiliary resonator is chosen to be one-third larger than that of the principal resonator, so that only every fourth mode of the principal resonator is hybridized.

In ring resonators, both single-pump parametric fluorescence and Bragg-scattering four-wave mixing effects are strongly enhanced by the presence of resonances that are otherwise not relevant to the desired dual-pump squeezing dynamics. These processes, and the associated resonances involved, are illustrated in Fig. 1a. To suppress these unwanted processes, it suffices to design a structure for which the two resonances labeled X1 and X2 are removed or suitably corrupted, without significantly degrading the properties of the resonances used for the two pumps and the signal, labeled P1 and P2, and S, respectively. To that end, a photonic molecule based on a two-resonator structure was designed. As shown in Fig. 1b, by tuning the auxiliary ring such that the X1 and X2 resonances of the principal ring nearly coincide in frequency with resonances of the auxiliary resonator, two new hybridized resonances emerge, strongly split and detuned from their original frequencies. The unwanted parametric processes involving the original X1 and X2 resonances are thereby highly suppressed. Since this modification to the X1 and X2 resonances can occur without having a significant impact on the P1, P2, and S resonances, strong enhancement of the desired squeezing process is maintained. This enables degenerate squeezing to be produced without degradation from unwanted single-pump parametric fluorescence and Bragg-scattering four-wave mixing, and without compromising the pump power efficiency of the source; more detail is available in Supplementary Notes 1 and 2. Similar multi-resonator schemes have been used to enable unidirectional frequency conversion^28^ and dispersion compensation^27^. In contrast to earlier work^12^ that used a second ring resonator to tune the effective escape efficiency of a device for nondegenerate bright squeezing, our device enhances degenerate quadrature squeezing by selectively suppressing unwanted processes.

## Results and discussion

The device was fabricated on a commercially available stoichiometric silicon nitride strip waveguide platform offered by Ligentec SA. The Si_3_N_4_ waveguide cross-section is 1500 nm × 800 nm (width × thickness) and is fully clad in SiO_2_. This platform and cross-section were selected for low propagation loss, lack of two-photon absorption, and high third-order optical nonlinearity. Independent microheaters were overlaid to provide thermal tuning of each resonator. The principal resonator was designed to have radius *R* = 114 μm, and the auxiliary resonator to have radius 0.75 × *R*, leading to free spectral ranges of 200 GHz for the principal resonator, and 267 GHz (approximately one-third larger) for the auxiliary resonator. The principal resonator is strongly over-coupled to the bus waveguide, resulting in an escape efficiency of ~90% in the wavelength range of interest; such over-coupling is important to limit the amount of loss experienced by the squeezed light on-chip.

**Fig. 2:**
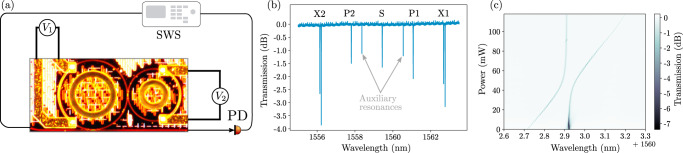
Linear characterization. **a** Micrograph of the photonic molecule structure and simplified schematic of the apparatus for linear characterization. The principal resonator is on the left, coupled at the bottom to a bus waveguide. The smaller ring on the right acts as the auxiliary resonator. Microheaters overlaid apply voltages *V*_1_ and *V*_2_ to the principal and auxiliary heaters, respectively. A swept wavelength source (SWS) and photodiode (PD) measure the transmission spectrum of the device. **b** Measured TE polarization transmission spectrum of the device in the wavelength range of interest, with resonators tuned to hybridize the unwanted resonances X1 and X2. Also evident are two resonances of the auxiliary resonator, which are indirectly weakly coupled to the bus waveguide via the principal resonator. **c** Transmission spectrum near the X1 resonance doublet of the device as the power dissipated by the auxiliary microheater is scanned. The resonance doublet exhibits the classic "avoided crossing" behavior associated with a pair of hybridized modes of a photonic molecule.

A micrograph of the device and the measured linear transmission spectrum of the fundamental TE resonances in the wavelength range of interest are exhibited in Fig. 2a, b, respectively. The auxiliary resonator microheater was tuned to achieve spectral alignment of the resonances associated with the auxiliary and principal resonator, leading to the formation of hybrid, split resonances associated with the unwanted single-pump parametric fluorescence and Bragg-scattering four-wave mixing. Three resonances of the principal resonator are preserved, displaying un-split Lorentzian lineshapes with loaded quality factors of ~3 × 10^5^.

The transmission spectrum of the device for a range of different auxiliary microheater settings is plotted in Fig. 2c for wavelengths near the X1 resonance. As the auxiliary resonances are tuned, the resonance doublet exhibits the classic avoided crossing behavior of coupled modes in a photonic molecule as the coupling strength (in this case determined by the detuning between X1 resonances of the auxiliary and principal resonators) is varied.

**Fig. 3:**
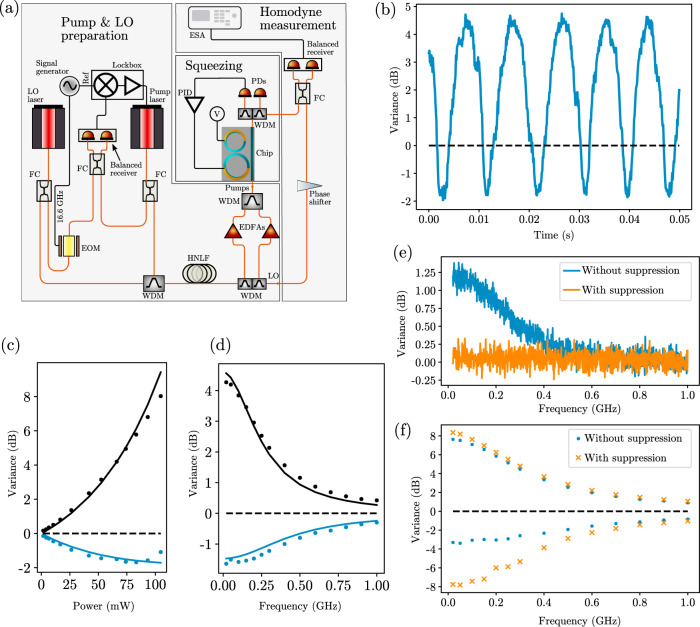
Squeezing experiment and results. **a** Simplified diagram of experimental setup used for squeezing measurements. Two lasers are phase-locked by monitoring the beat note generated when interfering them after phase-modulating one with a 16.6 GHz tone using a fast electro-optic modulator (EOM). These lasers serve as the local oscillator (LO) and first pump. They are then combined and coupled to a length of highly nonlinear fiber (HNLF) to generate a second pump, automatically phase-locked to the first pump and LO, via stimulated four-wave mixing. The two pumps are then separated from the LO, amplified by erbium-doped fiber amplifiers (EDFAs), and injected into the chip by edge couplers. The principal resonator of the photonic molecule is locked to the pump lasers' wavelengths by a slow proportional-integral-derivative (PID) loop, while the auxiliary resonator voltage supply (V) is set to maximize suppression of unwanted processes. Balanced homodyne detection is performed on the squeezed light output, as the LO phase is ramped. The quadrature variances are then recorded by an electrical spectrum analyzer (ESA). FC: fiber coupler; PD: photodiode; WDM: wavelength division multiplexing filter. More detail and a full experimental diagram are available in the Supplementary Note 1. **b** Quadrature variance at 20 MHz sideband frequency (blue trace) normalized to shot noise, plotted as a function of time as the LO phase is ramped. The black dashed line is the shot noise level. **c** Measured squeezing (blue points) and anti-squeezing (black points) at 20 MHz sideband frequency as the on-chip pump power is varied. Black dashed line is shot noise level; solid lines are a fit of the data points to a theoretical model. The three data points at the highest powers were omitted from the fit, as they are significantly affected by phase noise, which is not included in the model. **d** The squeezing (blue points) and anti-squeezing (black points) spectra, taken at 70 mW on-chip pump power. Black dashed line is shot noise level; Solid lines are a fit of the data points to a theoretical model. **e** Single-pump parametric fluorescence spectrum (normalized to shot noise) in the S mode, measured using homodyne detection with one pump turned off. The blue trace is taken with the auxiliary resonator tuned such that the P1, S, and X1 resonances are not significantly coupled to the principal resonator, effectively disabling the noise suppression, resulting in the contamination of the signal mode with broadband unwanted noise. The orange trace is taken with the auxiliary resonator tuned to split the X1 and X2 resonances (Fig. 2b), suppressing the noise to less than 0.1 dB above shot noise over the entire measurement band. For both traces the pump power was 75 mW, and the noise observed is phase-insensitive, as expected for single-pump parametric fluorescence. **f** On-chip squeezing and anti-squeezing spectra with noise suppression enabled (orange crosses) and disabled (blue points). The pump power was adjusted from 70 mW for the suppression-enabled case to 90 mW for the suppression-disabled case to keep the anti-squeezing approximately fixed, compensating for small changes in the overall four-wave mixing efficiency associated with tuning the auxiliary resonator. Squeezing is strongly diminished with noise suppression disabled.

A simplified diagram of the experiment to generate and measure squeezing is shown in Fig. 3a. Three phase- and frequency-locked beams were generated: two of these were amplified and filtered to serve as pumps, while the other beam was filtered and reserved for a use as a local oscillator (LO). The system used to generate the three beams—electro-optic frequency comb beat note locking, followed by stimulated four-wave mixing in highly nonlinear fiber—ensures that the local oscillator frequency is precisely equal to the average frequency of the pumps, and that the three optical phases are locked, as required for homodyne detection. One pump was tuned to the resonance near 1560.9 nm (the P1 resonance), and the principal resonator was actively locked to this pump to maximize the circulating pump power in the principal ring. The frequency of the other pump was tuned to a second resonance near 1557.7 nm (P2). The pump powers were set to be equal, and could be adjusted between 0 and 100 mW total power on-chip; pump powers reported in this work refer to the sum power of both pumps. The generated squeezed light was separated from the residual pumps and measured via balanced homodyne detection as the LO phase was ramped. Quadrature variances at sideband frequencies up to 1.0 GHz were recorded on an electrical spectrum analyzer. More details are available in the Methods section and Supplementary Note 1.

A representative quadrature variance trace at 20 MHz sideband frequency is shown in Fig. 3b as the LO phase is ramped. The directly measured squeezing was 1.65(1)dB. As the total collection and detection efficiency was 38(2)% (factoring in all losses experienced by the squeezed light except the resonator escape efficiency), this corresponds to ~8(1) dB of squeezing available at the device output on-chip. This is consistent with the maximum amount of squeezing possible from this device: as the principal resonator escape efficiency is ~90%, the maximum amount of squeezing on-chip is limited to 10 dB. Higher escape efficiencies, achieved by increasing the intrinsic quality factor of the device or increasing the coupling between the principal resonator and the bus waveguide would enable squeezing well in excess of 10 dB. For comparison, the level of squeezing required for fault-tolerant continuous variable quantum computation was recently shown to be ~10 dB^29^, and the record highest squeezing across all platforms is currently 15 dB from a hemilithic bulk-optical cavity^30^. Finally, we estimate the quantum state purity ${({V}_{\min }{V}_{\max })}^{-1/2}$^31^, where ${V}_{\min }$ and ${V}_{\max }$ are, respectively, the directly measured minimum and maximum quadrature variances of the 20 MHz sideband mode, to be ~0.7.

The measured squeezing and anti-squeezing at 20 MHz sideband frequency as a function of pump power are shown in Fig. 3c. Optimal squeezing was obtained with ~70 mW of total on-chip pump power, which is approximately half of the optical parametric oscillation (OPO) threshold. Beyond that power, the squeezing degrades as the OPO threshold is approached, possibly due to the finite phase stability of the LO and rapidly increasing variance of the anti-squeezed quadrature as the pump power is increased^32^. The squeezing is broadband: The squeezing and anti-squeezing spectra are exhibited in Fig. 3d, demonstrating that squeezing can be observed even for sideband frequencies up to 1 GHz. This is limited by the resonator linewidth and detection bandwidth.

To benchmark our results, the measured squeezing and anti-squeezing power scaling and spectra were fit to a theoretical model for degenerate squeezing in microring resonators with unwanted processes suppressed. These fits are exhibited by the solid lines in Fig. 3c, d, and show good agreement between theory and experiment. Independently measured values for the system loss, resonance linewidth, and pump detuning were used for these curves. In Fig. 3d, this leaves only the gain *g*, a dimensionless number proportional to the pump power and equal to unity at the OPO threshold, as the free parameter. This fit extracts *g* ≈ 0.46. The free parameter in the power scaling plot 3(c) is the proportionality constant that relates the gain *g* and the pump power; this fit predicts a gain *g* ≈ 0.50 at 70 mW, the pump power that optimizes squeezing. These independently extracted fit parameters are mutually consistent, and are also approximately consistent with our estimates for the OPO threshold being between 130 and 160 mW. This provides important validation for our model of the device, and further underscores the effectiveness of noise suppression in this system. More detail on the theoretical model and fitting procedure can be found in the Supplementary Note 3.

To assess the importance of the auxiliary resonator in suppressing unwanted processes, the parametric fluorescence noise spectrum generated in the S mode was measured with only the pump at P1 turned on, with 70 mW on-chip power. The results are shown in Fig. 3e for two different voltages applied to the auxiliary resonator. When the auxiliary resonator is tuned such that the X1 resonance is no longer hybridized, a strong noise contribution—more than 1.2 dB above shot noise—on all quadratures is observed. This excess noise is reduced to <0.1 dB above shot noise when the auxiliary resonator is tuned appropriately. In the absence of the auxiliary resonator, several dB of excess noise would therefore be present in the S mode, severely degrading the purity and achievable squeezing in the generated quantum state. This effect can also be directly seen in Fig. 3f, in which the on-chip squeezing and anti-squeezing spectra are shown with noise suppression enabled (orange crosses) and disabled (blue points). For fair comparison, the power was adjusted from 70 mW for the data with suppression enabled to 90 mW for the data with suppression disabled, in order to maintain a fixed degree of anti-squeezing. This adjustment in power was necessary to compensate for the small perturbations in the effective quality factors and resonance frequencies of the principal ring that arise from tuning the auxiliary resonator.

We have demonstrated degenerate quadrature squeezed vacuum with a noise reduction of 1.65(1) dB below shot noise, corresponding to 8(1) dB of squeezing available on-chip. The suppression of unwanted parametric processes was crucial to demonstrate strong single-mode squeezed light sources based on four-wave mixing. This was made possible by a designing a nanophotonic molecule to selectively suppress unwanted parasitic processes without significantly affecting squeezing efficiency. These results highlight the significant control that can be achieved over quantum nonlinear optical processes by exploiting nanophotonic platforms, and remove a significant barrier impeding progress towards scaling up devices for photonic quantum information processing.

## Methods

The chip was fabricated on a dedicated wafer run using a commercially available photolithographic process offered by Ligentec SA. The cross-section of the bus waveguide was 1000 nm × 800 nm (width x height), which is tapered up to 1500 nm width near the ring to match the its cross-section. While the desired dual-pump squeezing process would be optimized with normal dispersion, the ring waveguide cross-section resulted in weak anomalous dispersion; this was not an important factor, since the wavelength range used was relatively narrow. While self- and cross-phase modulation result in a pump power-dependent change in the effective dispersion of the principal resonator, their effects did not significantly hinder the device performance: the induced detunings were less than a half-linewidth in magnitude, and their effects were easily compensated for by a modest increase in pump power. The microheater placement was designed to minimize thermal cross-talk. The resulting wavelength tuning cross-talk is ~15%, i.e., a wavelength shift of *dλ* applied via the microheater on one ring results in a shift of about 0.15*dλ* in the other ring’s resonance spectrum. Edge couplers were used for coupling light in and out of the chip; ultrahigh numerical aperture (Nufern UHNA7) optical fibers were aligned to the chip facets, with index matching gel used to suppress reflections. The chip was temperature-stabilized using a thermoelectric cooler. The reported escape efficiency was extracted by individual least-squares fitting of the pump and signal resonances to a model for a lossy ring resonator. This resulted in escape efficiencies of 90% and 92% for the two pumps, and 91% for the signal resonance. Rounding to one significant figure, we report this as “~90%”. This value is consistent with the extinction ratios of the resonances evident in Fig. 2b.

Active optical components in Fig. 3a included: Continuous wave tunable diode lasers (Pure Photonics PPCL 550), a fiber-coupled fast electro-optic phase modulator (EOSpace), 200 m of highly nonlinear fiber (Sumitomo HNDS 1600BA-5), erbium-doped fiber amplifiers (Amonics AEDFA-33-B), and a fiber-coupled piezoelectric phase shifter (General Photonics FPS-001). Passive optical components included commercially available fiber-coupled taps, couplers, WDM filters, and polarization controllers. A multi-channel variable optical attenuator (OZ Optics) was used for controlling the powers of the pumps and LO. Optical isolators were placed before the nonlinear fiber, and before and after the chip to control back-reflection.

The electro-optic frequency comb locking used a commercially available lockbox (Vescent D2-135), and actuated the fast current input on one of the lasers. The 16.6 GHz tone was generated with a microwave signal generator (Syntotic DS-3000). The system used to generate the pumps and LO result in the relevant phase parameter 2*ϕ*_LO_ − *ϕ*_P1_ − *ϕ*_P2_ being stabilized to within 2 degrees root mean square, with *ϕ*_LO_, *ϕ*_P1_, and *ϕ*_P2_ the phases of the LO, P1, and P2, respectively. The slow proportional-integral-derivative (PID) loop used to lock the principal resonator to the pump lasers employed a field programmable gate array board (Red Pitaya) running PyRPL^33^.

Homodyne detection was carried out using a tunable fiber-coupled splitter (Newport) and a commercially available balanced receiver (Wieserlabs WL-BPD1GA, another copy also used for beat note detection in the phase locking loop). The quantum efficiency of the detectors was ~80%, and the bandwidth 1 GHz. The local oscillator power was set such that the detectors were operated well within the linear regime, with 14.7 dB of dark noise clearance at 20 MHz sideband frequency, gradually declining to 12 dB at 1 GHz; the full dark noise and shot noise spectra are available in Supplemntary Note 1. An electrical spectrum analyzer (Keysight NA9020A) was used to measure the photocurrent difference fluctuations, with the resolution bandwidth set to 1 MHz, video bandwidth 100 Hz. On-chip squeezing was inferred from measured squeezing by the formula *V*_on-chip_ = (*V*_meas_ + *η* − 1)/*η*, where *η* is the total collection efficiency after the ring output (including chip outcoupling loss, fiber component loss, and detector quantum efficiency), *V*_meas_ the measured minimum quadrature variance (relative to vacuum), and *V*_on-chip_ the inferred quadrature variance on-chip. The collection efficiency *η* was deduced by directly measuring, using classical light, the chip outcoupling loss and the loss in the fiber components after the chip. The detector quantum efficiency was calculated from the photodiode responsivity quoted by the manufacturer. The experimental uncertainty of 0.02 in *η* is propagated through the calculation of the on-chip squeezing to arrive at a final uncertainty of 1 dB in the 8 dB estimate.

## Supplementary information

Supplementary Information

## Data Availability

All data required to evaluate the conclusions of this work are available from the authors upon request.
