# Supplementary material for: Squeezed light from a nanophotonic molecule
Source: Nat Commun. 2021 Apr 14;12:2233. doi: 10.1038/s41467-021-22540-2 (PMC8046989; doi:10.1038/s41467-021-22540-2)
Supplement: Supplementary file 1 — Supplementary Information [file 41467_2021_22540_MOESM1_ESM.pdf]

Supplementary Information
Squeezed light from a nanophotonic molecule

Y. Zhang,[1] M. Menotti,[1] K. Tan,[1] V.D. Vaidya,[1] D.H. Mahler,[1]
L.G. Helt,[1] L. Zatti,[2] M. Liscidini,[2] B. Morrison,[1] and Z. Vernon[1,*]
[1]*Xanadu, Toronto, ON, M5G 2C8, Canada*
[2]*Department of Physics, University of Pavia, Via Bassi 6, 27100, Pavia, Italy*
(Dated: March 16, 2021)

I. SUPPLEMENTARY NOTE 1 – SQUEEZING EXPERIMENT

A full diagram of the experimental setup used for measuring squeezing is shown in Supplementary Figure 1. The apparatus can be divided into three functional blocks: Pump and local oscillator (LO) preparation, squeezing, and homodyne measurement. These are detailed in the following sections.

A. Pump and LO preparation

Homodyne detection of squeezed light generated from dual-pump four-wave mixing requires a bright LO beam with a phase ϕ_{LO} that is stable with respect to the sum phase $\phi_{\mathrm{P1}} + \phi_{\mathrm{P2}}$ of the pumps. That is, the phase parameter $2\phi_{\mathrm{LO}} - \phi_{\mathrm{P1}} - \phi_{\mathrm{P2}}$ must be stable, up to slow drifts that can be mitigated by a slow phase shifter. In addition, the LO frequency f_{LO} must precisely equal the average frequency $(f_{\mathrm{P1}} + f_{\mathrm{P2}})/2$ of the pumps. Finally, for stable operation, the frequency difference $f_{\mathrm{P1}} - f_{\mathrm{P2}}$ should be locked to a stable value.

To generate two pumps and an LO that satisfy these conditions, a hybrid radiofrequency (RF) electro-optic frequency comb locking and stimulated four-wave mixing scheme was employed. Two tunable diode lasers (Pure Photonics PPCL 550) generate the LO and first pump (P1) beams. A 10% fraction of the LO beam is tapped, aligned in polarization, and deeply phase-modulated by a fast electro-optic phase modulator (EOSpace) driven by a strong 16.6 GHz tone synthesized by RF signal generator (Syntotic DS-3000) and amplified by an RF amplifier (Minicircuits ZVE-3W-183+). The signal generator also outputs a 100 MHz reference tone, which is directed to a lockbox (Vescent D2-135) that includes a mixer and proportional-integral-derivative (PID) controller. The phase-modulated LO light is interfered on a 50:50 fiber coupler with a 1% fraction of the P1 light tapped off by a fiber coupler. The outputs of the 50:50 coupler are measured by a fiber-coupled balanced receiver (Wieserlabs WL-BPD1GA), with the electronic signal directed to the lockbox. In the lockbox, the 800 MHz beat note between the P1 beam and the 12th-order sideband of the phase-modulated LO beam is mixed down and beat against the 100 MHz reference, yielding a feedback signal directed to the fast current input of the P1 laser. This loop locks the optical frequencies of the LO and P1 lasers. While not necessary for this application, it also provides a degree of phase locking between the P1 pump and LO beams (to within approximately 12 degrees root-mean-square).

With the frequency locking loop persistently active, the remaining P1 and LO light are aligned in polarization and combined by a wavelength division multiplexing (WDM) filter and passed through an optical isolator (General Photonics) to limit back-reflections. With about 40 mW of power present at each wavelength, the light was then propagated through 200 m of highly nonlinear optical fiber (HNLF, Sumitomo HNDS 1600BA-5). Stimulated four-wave mixing in this fiber generated a bright beam with frequency $f_{\mathrm{P2}} = 2f_{\mathrm{LO}} - f_{\mathrm{P1}}$ for the second pump P2. This nonlinear process also ensures that the phase $2\phi_{\mathrm{LO}} - \phi_{\mathrm{P1}} - \phi_{\mathrm{P2}}$ is precisely fixed, as required. About 0.3 mW of power at f_{P2} is generated and available at the end of the HNLF fiber spool.

After the HNLF, the LO and two pumps are separated by WDM filters. The pumps are individually amplified by a pair of erbium-doped fiber amplifiers (Amonics AEDFA-33-B). The pumps and LO are sent through separate channels of a multi-channel digital variable optical attenuator (VOA, Oz Optics) for independent power control. Each of the three beams are independently aligned in polarization, with the LO then directed to a fiber-coupled piezoelectric phase shifter (General Photonics FPS-001) driven by a slow signal generator (Wavestation 2052) and piezo driver (Piezo Drive PD200). The pumps are combined by a WDM filter, passed through another optical isolator, with their total power monitored by a 1% tap and slow photodiode.

* zach@xanadu.ai

B. Squeezing

The two pumps are injected into the chip via ultra-high numerical aperture (Nufern UHNA7) fiber and a waveguide edge coupler. Index matching gel is used between the fiber and chip to suppress reflections at the facet. The chip is mounted on a thermo-electric cooler (TEC), which stabilizes the chip temperature via slow active feedback control carried out by PID loop controller (Arroyo 5240). A voltage supply (V) is used to set the auxiliary ring microheater voltage. The chip was wire bonded to ensure stable electrical control.

The squeezed light and residual pump light is coupled out of the chip to a WDM filter, which separates the two pumps from the squeezed beam. The two pump outputs are monitored by a slow photodiode, with the remaining pump light used as a signal to lock the principal ring to the P1 pump frequency. A photodiode actively monitors the P1 transmission, with the signal directed to a Red Pitaya field programmable gate array (FPGA) board running PyRPL [1]. This implements a PID loop actuating the principal ring microheater, locking the principal resonator to the pump frequency.

The total collection and detection efficiency experienced by the squeezed light after it exits the resonator is 38%: 71% from the chip out-coupling, 67% from the fiber components after the chip, and 80% from the quantum efficiency of homodyne detector. We estimate our confidence in this to be within about ±2%. Accounting for the 90% resonator escape efficiency, the total system efficiency experienced by the squeezed light during and after generation is 34%. This is consistent with the degree of squeezing and anti-squeezing observed when the auxiliary resonator noise suppression is optimal.

C. Homodyne measurement

The squeezed light output from the chip is interfered with the LO on a tunable fiber coupler (Newport), which is adjusted to obtain a precise 50:50 splitting ratio. The LO phase was modulated with a 100 Hz triangle wave signal. The two outputs from the tunable fiber coupler are incident on another balanced receiver (Wieserlabs WL-BPD1GA). The receiver has 1 GHz bandwidth, 30 dB common mode rejection ratio, 3500 V/W transimpedance gain, and is AC coupled. The quantum efficiency of the detectors is approximately 80%. The dark noise and shot noise spectra from 20 MHz to 1 GHz are shown in Supplementary Figure 2(a); the dark noise clearance (i.e., shot noise normalized to dark noise) is plotted in Supplementary Figure 2(b). At the 20 MHz sideband frequency used for the squeezing data in Fig. 3(b) and 3(c) of the main text, the clearance is 14.7 dB; this gradually declines to 12.0 dB at 1 GHz. The LO power used was 12 mW. The output signal from the receiver was connected to an electrical spectrum analyzer (ESA, Keysight NA9020A) with set to resolution bandwidth 1 MHz, video bandwidth 100 Hz.

II. SUPPLEMENTARY NOTE 2 – SUPPRESSION OF PARASITIC PROCESSES

The dual-pump scheme shown in Fig. 1(a) of the main text leads to a number of different processes associated with the $\chi^{(3)}$ nonlinear interaction. These include dual-pump spontaneous four-wave mixing (DP-SFWM), which is the desired interaction for producing degenerate squeezed vacuum states, single-pump SFWM (SP-SFWM), which generates photon pairs with one photon in mode S and one photon in either mode X1 or X2, and Bragg-scattering FWM (BS-FWM), which transfers a photon between S and X1 or X2 as a photon is transferred between P1 and P2. Finally, the third-order nonlinearity affects the spectral position of the resonant frequencies of the structure through self-phase modulation (SPM) and cross-phase modulation (XPM). The nonlinear Hamiltonian describing these processes can be written as

$$H_{\rm NL} = H_{\rm DP-SFWM} + H_{\rm SPM} + H_{\rm XPM} + H_{\rm SP-SFWM} + H_{\rm BS-FWM}, \tag{1}$$

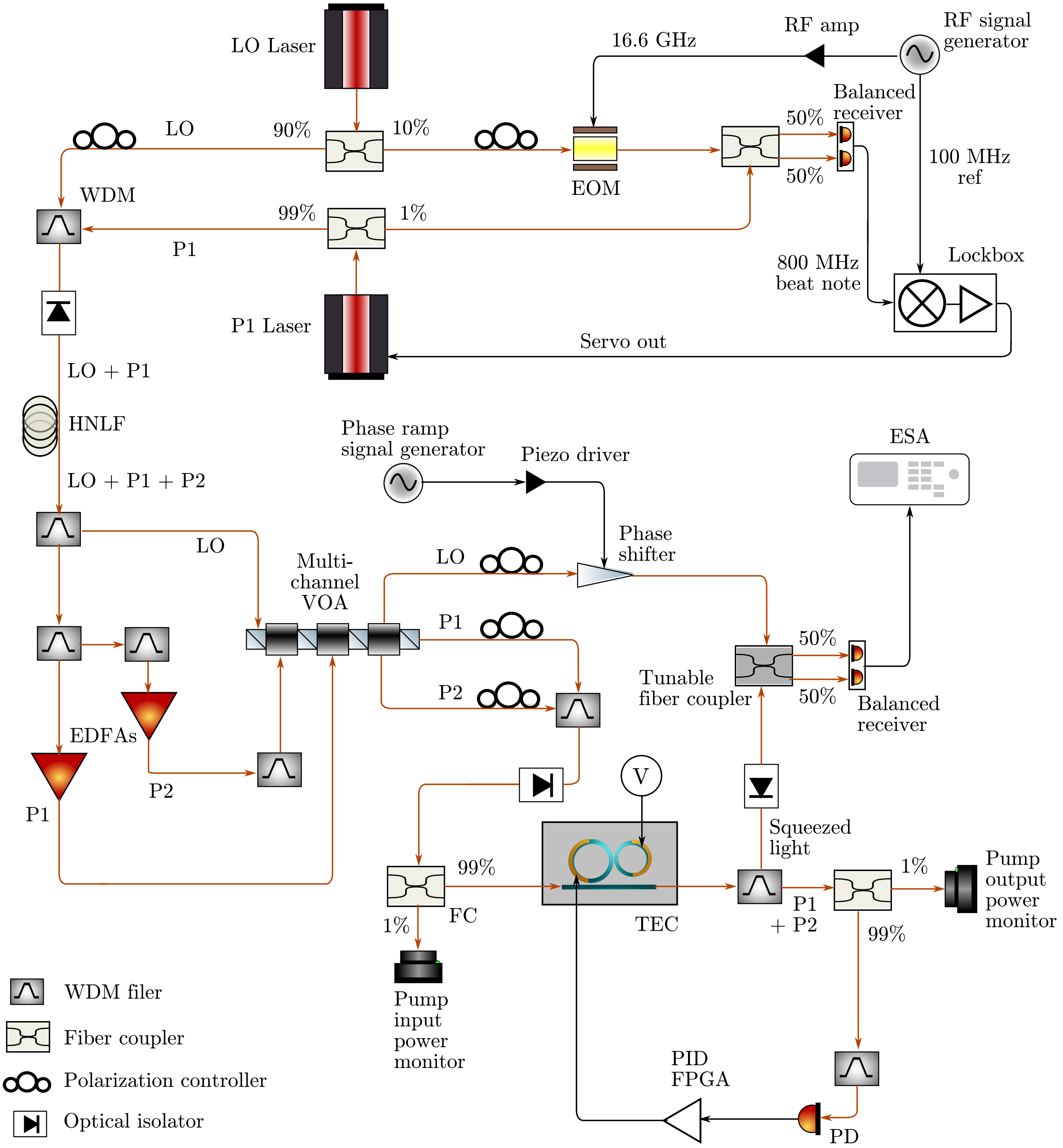

Supplementary Figure 1. Full schematic of experimental setup for measuring squeezing. Details in text.

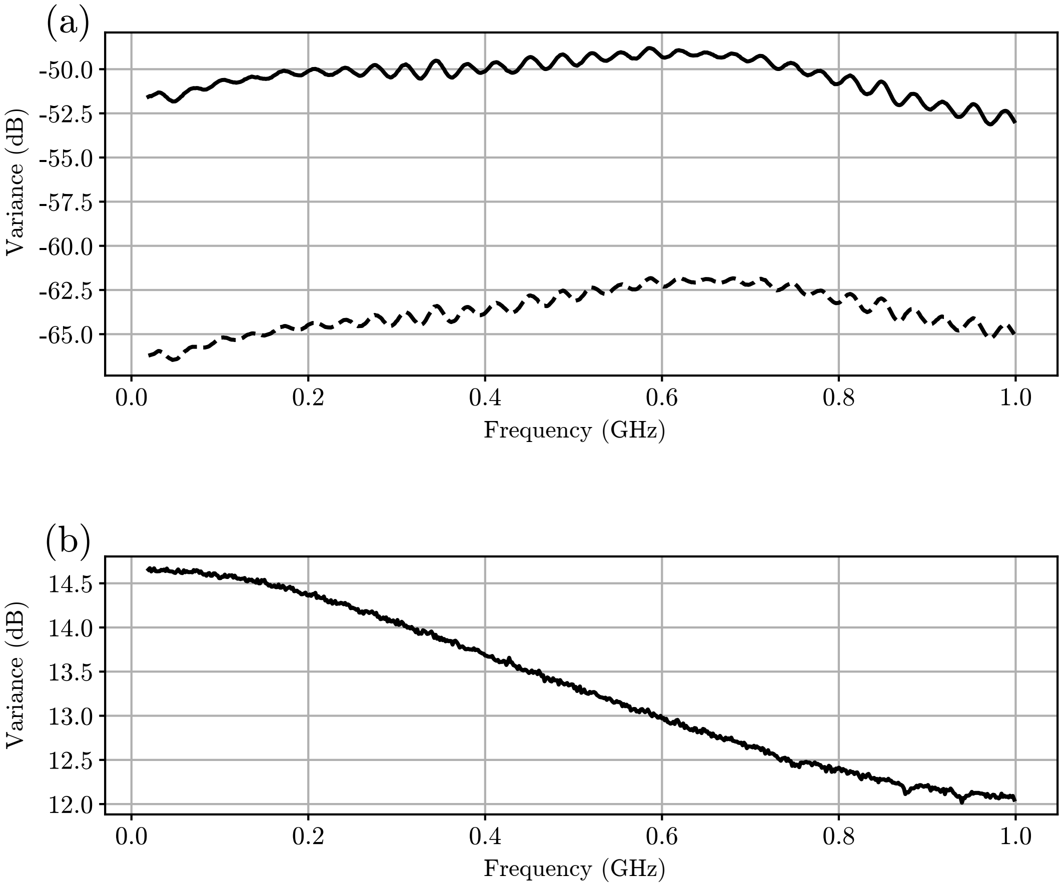

Supplementary Figure 2. (a) Dark noise (dashed line) and shot noise (solid line) spectra from 20 MHz to 1 GHz sideband frequencies. The shot noise trace is acquired with 12 mW LO power. (b) Noise clearance (shot noise normalized to dark noise) spectrum from 20 MHz to 1 GHz sideband frequencies for 12 mW LO power. The clearance varies from 14.7 dB at low frequencies to 12.0 dB at high frequencies.

where

$$H_{\mathrm{DP-SFWM}} = -\hbar\Lambda\left(b_{\mathrm{S}}^{\dagger}b_{\mathrm{S}}^{\dagger}b_{\mathrm{P1}}b_{\mathrm{P2}} + \mathrm{H.c.}\right), \tag{2}$$

$$H_{\mathrm{SPM}} = -\hbar\frac{\Lambda}{2}(b_{\mathrm{P1}}^{\dagger}b_{\mathrm{P1}}^{\dagger}b_{\mathrm{P1}}b_{\mathrm{P1}} + b_{\mathrm{P2}}^{\dagger}b_{\mathrm{P2}}^{\dagger}b_{\mathrm{P2}}b_{\mathrm{P2}}), \tag{3}$$

$$\begin{aligned} H_{\mathrm{XPM}} = -2\hbar\Lambda\Big(& b_{\mathrm{S}}^{\dagger}b_{\mathrm{P1}}^{\dagger}b_{\mathrm{S}}b_{\mathrm{P1}} + b_{\mathrm{S}}^{\dagger}b_{\mathrm{P2}}^{\dagger}b_{\mathrm{S}}b_{\mathrm{P2}} + b_{\mathrm{P1}}^{\dagger}b_{\mathrm{P2}}^{\dagger}b_{\mathrm{P1}}b_{\mathrm{P2}} + b_{\mathrm{X1}}^{\dagger}b_{\mathrm{P1}}^{\dagger}b_{\mathrm{X1}}b_{\mathrm{P1}} \\ &+ b_{\mathrm{X1}}^{\dagger}b_{\mathrm{P2}}^{\dagger}b_{\mathrm{X1}}b_{\mathrm{P2}} + b_{\mathrm{X2}}^{\dagger}b_{\mathrm{P1}}^{\dagger}b_{\mathrm{X2}}b_{\mathrm{P1}} + b_{\mathrm{X2}}^{\dagger}b_{\mathrm{P2}}^{\dagger}b_{\mathrm{X2}}b_{\mathrm{P2}}\Big), \end{aligned} \tag{4}$$

$$H_{\mathrm{SP-SFWM}} = -\hbar\Lambda\left(b_{\mathrm{X1}}^{\dagger}b_{\mathrm{S}}^{\dagger}b_{\mathrm{P1}}b_{\mathrm{P1}} + b_{\mathrm{X2}}^{\dagger}b_{\mathrm{S}}^{\dagger}b_{\mathrm{P2}}b_{\mathrm{P2}}\right) + \mathrm{H.c.}, \tag{5}$$

$$H_{\mathrm{BS-FWM}} = -2\hbar\Lambda\left(b_{\mathrm{P1}}b_{\mathrm{P2}}^{\dagger}b_{\mathrm{S}}b_{\mathrm{X1}}^{\dagger} + b_{\mathrm{P1}}b_{\mathrm{P2}}^{\dagger}b_{\mathrm{X2}}b_{\mathrm{S}}^{\dagger}\right) + \mathrm{H.c.}, \tag{6}$$

in which Λ is a constant quantifying the strength of the nonlinear interaction, and b_{J} is the annihilation operator for mode J in the ring. Here we are interested in the SP-SFWM and BS-FWM terms, which lead to the generation or destruction of a single photon in the mode S, and thus are a source of noise in the generation of degenerate squeezing. In a single ring resonator, when the resonances are nearly equally spaced in frequency, these processes are an intrinsic source of noise, and their mitigation, for example by a properly selected pump detuning [2], is typically associated with a weakening of the desired nonlinear process of DP-SFWM as well.

The photonic molecule approach described in the main text aims at suppressing these parasitic processes by engineering the electromagnetic field enhancement in the structure, which is directly related to the process efficiency. This is done by engineering the resonance positions, in analogy to modifying the energy levels of a true molecule, by coupling a second resonator to the ring used for squeezing. This allows to achieve the splitting of the X1 and X2

resonances, detuning them well away from their original frequencies, and thereby suppressing the above-mentioned undesirable parasitic processes of SP-SFWM and BS-FWM.

One can estimate the efficacy of this strategy by considering the nonlinear Hamiltonian in terms of asymptotic fields [3], which are directly connected to the resonant electromagnetic field enhancement factors $\mathcal{F}(k)$. In this case, the generic four-photon Hamiltonian associated with any of DP-SFWM, SP-SFWM, and BS-FWM can be written as:

$$H_{\rm NL} = -\int {\rm d}k_1 {\rm d}k_2 {\rm d}k_3 {\rm d}k_4 S\left(k_1, k_2, k_3, k_4\right) a^\dagger_{k_1} a^\dagger_{k_2} a_{k_3} a_{k_4} + {\rm H.c.}, \tag{7}$$

where k is the wavevector, a_k the annihilation operator of the (continuum) field associated with k, and

$$S(k_1, k_2, k_3, k_4) = \frac{3\Omega\bar{\Gamma}^{(3)}}{8\epsilon_0\mathcal{A}}\sqrt{\frac{\hbar\omega_{k_1}\hbar\omega_{k_2}\hbar\omega_{k_3}\hbar\omega_{k_4}}{(2\pi)^4}}\mathcal{F}(k_1)\mathcal{F}(k_2)\mathcal{F}(k_3)\mathcal{F}(k_4), \tag{8}$$

where $\mathcal{A}$ is the effective area, Ω a proportionality constant, $\bar{\Gamma}^{(3)}$ the third-order susceptibility [4], and $\mathcal{F}(k_i)$ the field enhancement factor at $k_i = \omega_i n_{\rm eff}(\omega_i)/c$, with $n_{eff}(\omega_i)$ the effective index and c the speed of light.

It is useful to define the overall resonant field enhancement associated with the desired four-wave process

$$F(\omega_1, \omega_2, \omega_3, \omega_4) = \mathcal{F}(k(\omega_1))\mathcal{F}(k(\omega_2))\mathcal{F}(k(\omega_3))\mathcal{F}(k(\omega_4)), \tag{9}$$

which allows one to compare the strength of the various Hamiltonian terms for different structures and pumping configurations.

In Fig. 3 we plot $F_{\rm DP-SFWM}(\omega_{\rm S}, \omega_{\rm S}, \omega_{\rm P1}, \omega_{\rm P2})$ for DP-SFWM in our photonic molecule as a function of the field coupling coefficient κ between the generating ring and the auxiliary ring necessary to split the resonances at $\omega_{\rm X1}$ and $\omega_{\rm X2}$ associated with the parasitic processes. Here $\omega_{\rm P1}$ and $\omega_{\rm P2}$ are the pump frequencies and $\omega_{\rm S} = (\omega_{\rm P1} + \omega_{\rm P2})/2$ is that of the generated photons. The system has been designed such that all the three fields are resonantly coupled to the generating ring. As expected, the resonances at $\omega_{\rm P1}$, $\omega_{\rm P2}$, and $\omega_{\rm S}$ are only very weakly dependent on κ in position and quality factor, thus $F_{\rm DP-SFWM}(\omega_{\rm S}, \omega_{\rm S}, \omega_{\rm P1}, \omega_{\rm P2})$ remains nearly constant.

In Supplementary Figure 3 we plot also the ratio

$$\mathcal{R} = \frac{F_{\rm DP-SFWM}(\omega_{\rm S}, \omega_{\rm S}, \omega_{\rm P1}, \omega_{\rm P2})}{F_{\rm SP-SFWM}(\omega_{\rm S}, \omega_{\rm X1}, \omega_{\rm P1}, \omega_{\rm P1})}, \tag{10}$$

which allows one to estimate the relative suppression of the parasitic process associated with the generation of one photon at $\omega_{\rm S}$ and one photon at $\omega_{\rm X1}$ due to SP-SFWM associated with the pump at $\omega_{\rm P1}$. A similar result could be shown for SP-SFWM involving the resonance at $\omega_{\rm X2}$ or BS-FWM. One can see that as a result of the splitting of the resonance at $\omega_{\rm X1}$ the overall field enhancement for the parasitic process decreases with κ and is attenuated by about a factor of 60 (relative to the enhancement maintained by the desired DP-SFWM process) at our operating point. Achieving the same result by symmetrically detuning the pumps from their respective resonances (the approach taken by Zhao et al. [2]) would be possible, but it would also result in decreasing the desired field enhancement $F_{\rm DP-SFWM}(\omega_{\rm S}, \omega_{\rm S}, \omega_{\rm P1}, \omega_{\rm P2})$ by three orders of magnitude compared to the value obtained with our photonic molecule.

It is also important to consider whether other four-wave mixing processes, involving resonances outside those considered in this discussion, could contribute noise to the squeezing band. The leading contribution from such processes involves cascaded stimulated and spontaneous four-wave mixing: classical stimulated four-wave mixing leads to the generation of coherent light at a pair of resonances with frequencies three free spectral ranges above and below the S resonance. These stimulated waves are weak compared to the pumps, having at most a few mW of generated light for the pump powers used, but they are strong enough to warrant concern over the corresponding spontaneous four-wave mixing processes that could add noise photons to the squeezing band.

The single-pump processes involving light generated in the stimulated modes are suppressed by the auxiliary resonator. The ring radius of that resonator is chosen to hybridize every fourth resonance of the principal resonator, and it accomplishes this over a very wide span of wavelengths. Thus the resonances lying six free spectral ranges above and below the S resonance are also strongly hybridized (i.e., split and detuned). These resonances are involved in the single-pump spontaneous processes that would add photons to the S mode via SP-SFWM arising from the stimulated waves acting as pumps. Therefore such processes are similarly suppressed in our device.

However, dual-pump processes involving spontaneous four-wave mixing driven by one of the bright pumps, and one of the stimulated waves, is not suppressed by the auxiliary resonator. These processes are much weaker than

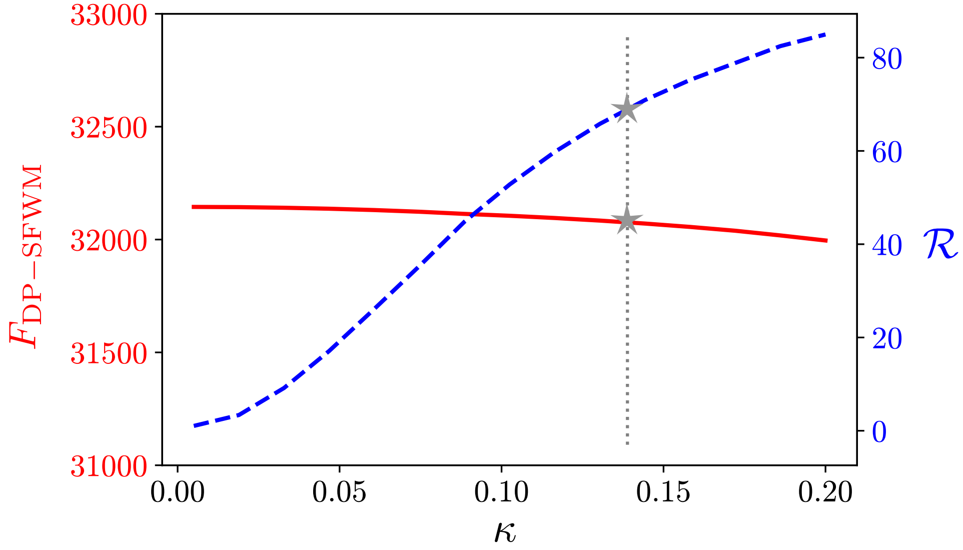

Supplementary Figure 3. Field enhancement $F_{\mathrm{DP-SFWM}}$ associated with the desired DP-SFWM process in the photonic molecule structure (red solid line, left vertical axis), and relative noise suppression ratio $\mathcal{R}$ (blue dashed line, right vertical axis) as a function of the coupling κ between the principal and auxiliary ring. The dashed grey line indicates the approximate coupling chosen for the device reported in the main text; grey star markers indicate the operational point obtained for field enhancement and relative suppression.

the other spontaneous processes discussed so far, since they involve one of the weaker, stimulated waves acting as a pump; they are automatically suppressed by approximately the ratio between the power in the generated stimulated waves and the power in the input pumps. In our experiments, this ratio is on the order of 10^{-3} for the pump powers that optimize squeezing, and thus about 30 dB of suppression for this process is automatically ensured. This could be improved by the addition of extra rings specifically designed to suppress stimulated processes.

A complete accounting of every four-wave mixing process possible in this system is beyond the scope of this work. A full discussion and analysis is left to future studies.

III. SUPPLEMENTARY NOTE 3 – THEORY OF DEGENERATE SQUEEZING

To model the generation of degenerate squeezing in a microring resonator system for which noise suppression has been implemented, we model the intra-resonator dynamics using a set of coupled mode equations for the two pump resonances and the S resonance. We follow the same treatment used for calculating nondegenerate quadrature squeezing in the Supplementary Materials section S1 of Vaidya *et al.* [5]; here we present a summary of this calculation adapted to the degenerate case.

Treating the pumps classically, we represent their steady state intra-resonator amplitudes as $\overline{\beta}_{\mathrm{P1}}e^{-i(\omega_{\mathrm{P1}}-\Delta_{\mathrm{P1}})t}$ and $\overline{\beta}_{\mathrm{P2}}e^{-i(\omega_{\mathrm{P2}}-\Delta_{\mathrm{P2}})t}$, where Δ_{P1} and Δ_{P2} are the detunings of each pump from resonance, and $\overline{\beta}_{\mathrm{P1}}$ and $\overline{\beta}_{\mathrm{P2}}$ are constants. The equation of motion for the S resonance mode is then

$$\left(\frac{d}{dt}+\overline{\Gamma}_{\mathrm{S}}+i\omega_{\mathrm{S}}-2i\Lambda(|\beta_{\mathrm{P1}}|^2+|\beta_{\mathrm{P2}}|^2)\right)b_{\mathrm{S}}(t)=-i\gamma_{\mathrm{S}}^*\psi_{\mathrm{S}<}(0,t)-i\mu_{\mathrm{S}}^*\phi_{\mathrm{S}<}(0,t)+2i\Lambda b_{\mathrm{S}}^\dagger(t)\overline{\beta}_{\mathrm{P1}}\overline{\beta}_{\mathrm{P2}}e^{-i(\omega_{\mathrm{P1}}+\omega_{\mathrm{P2}}-\Delta_{\mathrm{P1}}-\Delta_{\mathrm{P2}})t}, \tag{11}$$

where $\overline{\Gamma}_{\mathrm{S}}$ is the damping rate of the S resonance, related to the loaded quality factor Q_{S} via $Q_{\mathrm{S}}=\omega_{\mathrm{S}}/2\overline{\Gamma}_{\mathrm{S}}$, and γ_{S} and μ_{S} are the coupling coefficients to the input channel vacuum field and loss channel vacuum field $\psi_{\mathrm{S}<}(0,t)$ and $\phi_{\mathrm{S}<}(0,t)$, respectively.

The squeezing spectrum $S(\Omega)$ can be calculated via the relation

$$S(\Omega)=1+N(\Omega,\Omega)+N(-\Omega,-\Omega)+2\mathrm{Re}\{e^{-2i\phi_{\mathrm{LO}}}M(\Omega,-\Omega)\}, \tag{12}$$

where ϕ_{LO} is the local oscillator phase and the moments M and N are defined via

$$M(\Omega,\Omega')\delta(\Omega-\Omega')=v_S\langle\psi_{\mathrm{S}>}(\Omega)\psi_{\mathrm{S}>}(\Omega')\rangle \tag{13}$$

and

$$N(\Omega,\Omega')\delta(\Omega-\Omega')=v_S\langle\psi_{\mathrm{S}>}^\dagger(\Omega)\psi_{\mathrm{S}>}(\Omega')\rangle, \tag{14}$$

where $\psi_{\mathrm{S}>}(\Omega)$ is the output channel field at sideband frequency Ω. Calculating these quantities from the solution obtained to Supplementary Equation 11, we obtain

$$S_\pm(\Omega)=1+\frac{4\eta g\left(2g\pm\sqrt{\left(\frac{\Omega^2}{\overline{\Gamma}_{\mathrm{S}}^2}+1+g^2-\frac{\Delta^2}{\overline{\Gamma}_{\mathrm{S}}^2}\right)^2+4\frac{\Delta^2}{\overline{\Gamma}_{\mathrm{S}}^2}}\right)}{\left(\frac{\Omega^2}{\overline{\Gamma}_{\mathrm{S}}^2}-1+g^2-\frac{\Delta^2}{\overline{\Gamma}_{\mathrm{S}}^2}\right)^2+4\frac{\Omega^2}{\overline{\Gamma}_{\mathrm{S}}^2}}, \tag{15}$$

where $S_+(\Omega)$ and $S_-(\Omega)$ are respectively the maximum (over all quadrature angles) anti-squeezing and squeezing at sideband Ω, Δ is the overall net detuning associated with the degenerate spontaneous four-wave mixing process, $\Delta=2\Lambda(|\beta_{\mathrm{P1}}|^2+|\beta_{\mathrm{P2}}|^2)-(\Delta_{\mathrm{P1}}+\Delta_{\mathrm{P2}})/2$ including both pump detuning and XPM-induced detuning, η is the overall total system transmission efficiency including the ring escape efficiency and collection and detection efficiencies, and g is a dimensionless gain parameter defined as

$$g=\frac{2\Lambda|\beta_{\mathrm{P1}}\beta_{\mathrm{P2}}|}{\overline{\Gamma}_{\mathrm{S}}}. \tag{16}$$

In this treatment, the OPO threshold corresponds to a gain parameter $g=1$.

The expression in Supplementary Equation 15 is used to fit the experimental squeezing spectrum traces in Figs. 3(c-d) of the main text. The independently measured parameters for $\eta=0.34$, $\overline{\Gamma}_{\mathrm{S}}=\omega_{\mathrm{S}}/2Q_{\mathrm{S}}\approx 2.0\times10^9\mathrm{s}^{-1}$, and $\Delta=0.15\times\overline{\Gamma}_{\mathrm{S}}$ were used to fit 3(d), with g kept as the free fitting parameter. The resulting best least-squares fit yielded $g\approx 0.46$, consistent with our estimate of the corresponding pump power being about half that of the OPO threshold. This fit was performed simultaneously to the squeezing and anti-squeezing data, i.e., the parameter g was constrained to be equal for both the maximum and minimum quadrature variance data across the range of sidebands in the spectrum. The fit for the data in Fig. 3(c) used the same parameters and $\Omega=2\pi\times 20$ MHz, and assumed a linear relationship between the total pump power and g, consistent with the definition of Supplementary Equation 16. The proportionality coefficient between g and the input power was kept as the free fitting parameter. With this form, the fit predicted the gain parameter at the pump power that optimized squeezing to be $g\approx 0.50$, again consistent with our estimate of the OPO threshold and the result of the fit to the squeezing spectra in Fig. 3(d). The three data points at the highest powers were omitted from the fit in Fig. 3(c), as they are significantly affected by phase

noise, which is not included in the model. Inclusion of these points does not significantly change the extracted fit parameters. Finally, it should be noted that the exact OPO threshold depends on other factors not included in this model, such as dispersion which would give rise to different net effective detunings. The consistency between the extracted fit parameters and the estimated OPO threshold is therefore not an especially precise statement; that the fit parameters independently extracted from the two fits are consistent is a more significant validation of our model.

[1] L. Neuhaus, R. Metzdorff, S. Chua, T. Jacqmin, T. Briant, A. Heidmann, P.-F. Cohadon, and S. Deléglise, PyRPL (Python Red Pitaya Lockbox)-an open-source software package for FPGA-controlled quantum optics experiments, in *European Quantum Electronics Conference* (Optical Society of America, 2017) p. EA_P_8.

[2] Y. Zhao, Y. Okawachi, J. K. Jang, X. Ji, M. Lipson, and A. L. Gaeta, Near-degenerate quadrature-squeezed vacuum generation on a silicon-nitride chip, Physical Review Letters **124**, 193601 (2020).

[3] M. Liscidini, L. G. Helt, and J. E. Sipe, Asymptotic fields for a hamiltonian treatment of nonlinear electromagnetic phenomena, Physical Review A **85**, 013833 (2012).

[4] N. A. R. Bhat and J. E. Sipe, Hamiltonian treatment of the electromagnetic field in dispersive and absorptive structured media, Physical Review A **73**, 063808 (2006).

[5] V. D. Vaidya, B. Morrison, L. G. Helt, R. Shahrokshahi, D. H. Mahler, M. J. Collins, K. Tan, J. Lavoie, A. Repingon, M. Menotti, N. Quesada, R. C. Pooser, A. E. Lita, T. Gerrits, S. W. Nam, and Z. Vernon, Broadband quadrature-squeezed vacuum and nonclassical photon number correlations from a nanophotonic device, Science Advances **6**, eaba9186 (2020).
